# Influence of Unidirectional Vacuum Application on Bone Healing in Maxillofacial Surgery

**DOI:** 10.3390/cells14100751

**Published:** 2025-05-21

**Authors:** Tom Alexander Schröder, Athanasios Karasavvas, Maximilian Bauckloh, Matthias C. Schulz, Günter Lauer, Lysann Michaela Kroschwald

**Affiliations:** 1 Department of Oral and Maxillofacial Surgery, Faculty of Medicine “Carl Gustav Carus”, Technische Universität Dresden, Fetscherstraße 74, 01307 Dresden, Germany; 2 Department of Oral and Maxillofacial Surgery, University Hospital Tübingen, Eberhard Karls Universität Tübingen, Osianderstraße 2-8, 72076 Tübingen, Germany; 3 Centre for Translational Bone, Joint and Soft Tissue Research, University Hospital “Carl Gustav Carus”, Technische Universität Dresden, Fetscherstraße 74, 01307 Dresden, Germany

**Keywords:** VAC, NPWT, bone healing, regeneration, maxillofacial surgery

## Abstract

Negative-pressure wound therapy (NPWT) using vacuum-assisted closure (VAC) is a well known tissue defect bridging method that applies a vacuum pump to sterile, open-cell foam dressings via suction tubes. Although it has mostly been described for soft tissue use, there are also a few studies concerning its use on hard tissue. However, as oral and maxillofacial surgery has to deal with both soft and hard tissue, which lie next to each other in these regions, there is a particular need to assess the influence of negative pressure on bone. Therefore, the effects of different negative pressure levels (530 mbar and 725 mbar) and atmospheric pressure (1013 mbar) on bone tissue cultures and osteoblast cell cultures were investigated over periods of 1, 3, and 6 weeks. During the culture period, osteoblast growth and the tissue regeneration of bone defects were studied in vitro using tissue cultures that were histologically supplemented by cytological investigations and quantitative RNA expression studies. In the bone defect model, there was a faster defect reduction using NPWT; the effect was especially strong for 530 mbar. Compared to the control group, up to 30% more newly generated bone tissue was detected. This effect on the mineralization capacity was assessed by the mRNA expression of osteogenic marker genes, as well as the receptor activator of nuclear factor κB ligand (RANKL) and osteoprotegerin (OPG), two multifaceted cytokines that regulate bone metabolism. The influence of negative pressure consequently resulted in a decreased RANKL/OPG ratio in osteoblasts. Associated with the upregulation of marker genes to up to 400%, including Col1, BMP4, OCN, and RUNX2, the decrease in the RANKL/OPG ratio to 41% indicates the stimulation of osteogenesis. Since VAC has been shown to be a safe and effective method to close wounds in general, these data suggest that patients suffering from compound bone and soft tissue defects in the maxillofacial area may benefit from an adapted therapy approach accelerating both soft and hard tissue regeneration.

## 1. Introduction

Negative-pressure wound therapy (NPWT) using vacuum-assisted closure (VAC) devices is a well established therapy method used to accelerate the healing of acute and chronic wounds. It facilitates healing through changes in growth factor expression, micro- and macro-deformation, blood flow exudate removal, and bacterial load reduction within the wound bed [1].

VAC has become increasingly popular as a means of providing temporary or longer-term coverage for various wound types, e.g., complex traumatic wounds with extensive tissue damage and even with exposed bone; compartment syndrome after fasciotomy; infected surgical wounds; and chronical wounds like diabetic foot ulcers and venous stasis ulcers [2,3]. Any type of tissue can be covered, including blood vessels, bone, synthetic mesh, and even hardware [4,5,6]. Particularly when managing traumatic injuries, the use of VAC coverage or direct closure depends on the degree of damage to the associated tissue structures. It was Fleischmann et al. who first described the use of NPWT to treat wounds with open fractures [7]. Soon after, the first VAC device was developed by Argenta et al. [8].

As a temporary bridging method that can be used until definitive wound closure is performed, the technique is based on covering the tissue defect with a sterile, open-cell foam or gauze dressing connected to a vacuum pump through suction tubes [9]. Generally, negative pressure of up to 125 mmHg is applied either in a constant or intermittent mode. With the intermittent method, Morykwas et al. showed increased granulation tissue formation compared to constant or none-NPWT therapy [10,11]. Three main mechanisms are believed to be involved. The first mechanism is the four-fold-increased blood flow using intermittent sub-atmospheric pressure (5 min on and 2 min off) [10,12]. The second mechanism pertains to the mechanical stress on the cells increasing their proliferation, enhancing mitotic activity [13,14]. The third mechanism is the suction itself, which removes wound healing inhibitors like metalloproteases [2] while also reducing bacterial counts [10,15]. Therefore, deep infections and chronic wounds can also be successfully treated with VAC [15]. A reduction in the risk of infection and an accelerated wound healing process have been reported in a number of systematic reviews [16,17]. Due to rapid wound granulation [18], at our clinic, we apply intermittent VAC at the donor site after harvesting the fibula for the reconstruction of the mandible after ablative tumor surgery. However, there are only a few studies on the application of negative-pressure wound therapy in oral and maxillofacial surgery. The complex anatomical shape of this region of the body, as well as the composition of soft and hard tissue, may be reasons for its limited use.

Although a combination of VAC and open bone grafting has been applied to treat patients with osteomyelitis or bone non-union to achieve an optimal curative effect [19,20], few studies focus on hard tissue regeneration using VAC therapy. Thus, this study aims to fill the gap in knowledge concerning the effect of negative pressure on bone regeneration. Therefore, in this study, a bone defect tissue culture model and osteoblast cell cultures generated from cylindrical bone specimens of pig mandibles were exposed to the influence of negative pressure (530 mbar and 725 mbar) to investigate bone healing and osteoblast growth and differentiation in vitro.

## 2. Materials and Methods

### 2.1. Tissue Preparation and Processing

**Figure 1:**
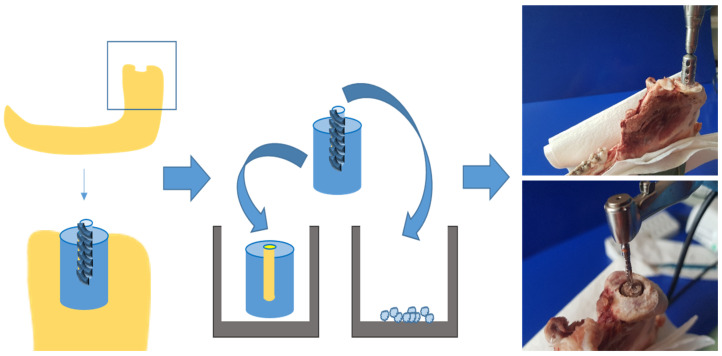
Generating bone cylinders from lower jaw of pigs by using a trepan drill bit and an implant drill bit.

In order to assess the effect of negative pressure on bone tissue growth and on osteoblast proliferation and differentiation, organ cultures of bone cylinders, as well as osteoblast cell cultures, were established using bone from pigs’ mandibular condylar processes (6–8 months old; see Figure 1). Mandibles were retrieved from a local slaughterhouse within 24 h of slaughtering. After removing the perichondrium, identical bone cylinders (diameter 5 mm, depth 10 mm) were cut out of the heads of the mandibular condylar processes samples (see Figure 1) using a steel trepan drill bit (Ø 5 mm; Helmut Zepf Medizintechnik GmbH, Seitingen-Oberflacht, Germany; see Figure 1). To create an identical bone defect (diameter 2.8 mm, depth 5 mm) in each cylinder, a central borehole was drilled with an implant drill bit (Straumann AG, Basel, Switzerland). The bony borehole grindings were collected (see Figure 1), quantified, and cultivated.

### 2.2. Organ Culture of Bone Cylinders with Central Defect

The bone cylinders were briefly washed in culture medium and then placed into 6-well plates (see Figure 1) containing Dulbecco’s modified Eagle’s medium (DMEM) supplemented with 10% fetal calf serum (FCS) and 1% amphotericin/penicillin/streptomycin (all Gibco, Eggenstein, Germany) and cultured at 37 °C and 5% CO_2_ (see Figure 1). The culture medium was changed twice per week. Samples were incubated for up to 6 weeks and treated with negative pressure (530 mbar and 725 mbar) every 12 h for 30 min, compared to the control group (1013 mbar). Negative pressure was generated using a vacuum pumping unit (PC 3004 Vario, Vacuubrand GmbH Co., KG; Wertheim, Germany).

### 2.3. Osteoblast Cell Cultures from Bony Borehole Grindings

After 24 h of re-equilibration in DMEM under standard culture conditions, bone borehole grindings were seeded into 6-well plates containing DMEM supplemented with 15% FCS, 1% L-glutamate, and 1% penicillin/streptomycin (all Gibco, Eggenstein, Germany) to establish primary porcine osteoblast cultures. After two passages for cell expansion, osteoblasts were seeded at a density of 1 × 10^6^ cells per well and incubated for up to 6 weeks. The medium was replaced twice per week. Osteoblast cultures were exposed to negative pressure (530 mbar and 725 mbar) as described above.

### 2.4. Histological Preparation and Characterization

After 1, 3, or 6 weeks of incubation, the bone cylinders were fixed in 4% neutral buffered formalin for at least 7 days, followed by washing and dehydration in a graded series of alcohol solutions with increasing concentrations up to 100%. For embedding, methyl methacrylate (Technovit 9100 New, Kulzer GmbH, Wehrheim, Germany) was used according to the manufacturer’s instructions.

**Figure 2:**
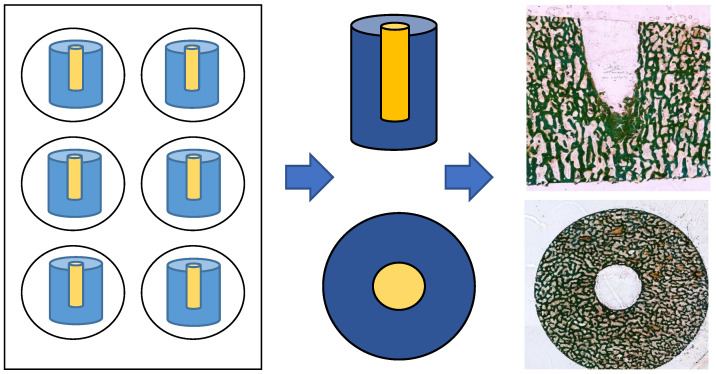
Preparation of cylindical specimen: cross-section and longitudinal sections after Masson’s Goldner staining.

The defect zone was sectioned into 80 µm thick slices (both the cross-section and longitudinal section) using an abrasive cutoff machine (EXAKT 400 CS, EXAKT Advanced Technologies GmbH, Norderstedt, Germany). Methyl methacrylate was removed by treating the sections with xylene (VWR International GmbH, Darmstadt, Germany) for 20 min each, followed by two treatments with 2-methoxyethyl acetate for 20 min each, before a final treatment with 96% ethanol. Samples were rehydrated in a graded series of ethanol corresponding to the ethanol concentration of the first staining solution. The sections were then stained for light microscopy (Leica DMRBE Research Microscope, Camera Leica DC300, Leica Microsystems, Wetzlar, Germany) using Masson’s Goldner staining (Morphisto, Offenbach am Main, Germany; see Figure 2).

Nuclei were stained for 15 min with hematoxylin according to Weigert (iron-hematoxylin-Kit, VWR, International GmbH, Darmstadt, Germany), followed by azophloxin staining for 15 min. Samples were then washed in 1% acetic acid and placed in acid orange G solution. After rinsing in 1% acetic acid for 30 s, the sections were stained with light green for 5 min and subsequently rinsed again in 1% acetic acid for 5 min (all Masson-Goldner trichrome staining kit, Merck KGaA, Darmstadt, Germany).

To evaluate tissue growth and regeneration within the central bone defect, bone cylinders prepared for histology were examined using an Olympus BX 61 microscope (Olympus Deutschland GmbH, Hamburg, Germany) and cell F Imaging Software (version 2008) for Life Science (Olympus). The histological analysis focused on the structure of bone and soft tissue in the defect area. For histomorphometry, the remaining width of the defect was measured between the cranial and caudal margins and compared to the initial defect width at identical positions. The original defect borders and those after new bone formation were easily distinguishable due to differences in bone morphology. Additionally, the amount of newly formed bone within the entire defect area was quantified and expressed as a percentage. Histomorphometric calculations were performed as described before [21].

### 2.5. Fluorescence Microscopy, Proliferation, and Differentiation Analyses on Osteoblast Cultures

For fluorescence microscopy analyses, cells were fixed in 4% formaldehyde. The actin cytoskeleton and cell nuclei were stained with AlexaFlour 488^®^ phalloidin (Invitrogen, Carlsbad, CA, USA) and DAPI (Sigma Aldrich, Taufkirchen, Germany), respectively. Imaging was performed using a Keyence BZ9000E fluorescence microscope (Keyence, Neu-Isenburg, Germany).

Cell proliferation was assessed by measuring the increase in DNA content at different time points. A calibration curve was used to convert DNA content to cell number. DNA quantification was performed using the Quantifluor assay (Promega, Madison, WI, USA).

Alkaline phosphatase (ALP) activity was measured using 4-nitrophenyl phosphate disodium salt hexahydrate (Sigma Aldrich, Taufkirchen, Germany) as a substrate, with a calibration curve generated using p-nitrophenol (Sigma Aldrich, Taufkirchen, Germany). Absorbance was determined at 405 nm.

Calcium deposition was quantified in cell lysates using the Fluitest^®^ Ca-CPC at 590 nm after the addition of 6 M HCl (ratio 1:24). Both ALP activity and calcium deposition were normalized to cell number as determined by DNA quantification. All measurements were performed using a spectrofluorometer (Infinite M200pro; Tecan Trading AG, Männedorf, Switzerland).

### 2.6. RNA Isolation and Quantification

RNA was extracted using the RNeasy^®^ Plus Mini Kit (Qiagen, Hilden, Germany), following the manufacturer’s instructions. The RNA was dissolved in 20 µL RNase-free water (Invitrogen, Carlsbad, CA, USA), and concentrations were measured with an Infinite^®^ 200 Pro plate reader (Tecan, Männedorf, Switzerland). Samples with a 260/280 ratio of ≥2 were considered pure and used for further analysis.

Reverse transcription was performed using 0.5 µg of template RNA and random hexamer primers with the iScript cDNA Synthesis Kit (Bio-Rad Lab, Munich, Germany). Quantitative real-time PCR was carried out in 20 μL reaction volumes containing 0.5 ng/μL diluted cDNA, 10 μL iQ SYBR Green Supermix with Taq polymerase (Bio-Rad Laboratories, Munich, Germany), 0.6 μL of 10 pmol/µL forward primer, and 0.6 μL of 10 pmol/µL reverse primer. Amplification was performed on a Stratagene MX300P cycler (SABiosciences, Qiagen, Hilden, Germany) under the following conditions: 95 °C for 3 min, followed by 40 cycles of 95 °C for 15 s, 60 °C for 30 s, and 72 °C for 30 s, with a final cycle of 55 °C for 1 min and 95 °C for 30 s. The results were calculated using the ΔΔCT method and are presented as fold changes relative to GAPDH expression. Primers were purchased from Eurofins Genomics Germany GmbH (Ebersberg, Germany) according to the sequences published by Lüthje et al. in 2018 [22].

### 2.7. Statistical Analyses

Statistical analyses at defined incubation time points were performed using one-way analysis of variance (ANOVA) with Bonferroni adjustment of *p*-values to evaluate the effects of VAC-treated samples compared to untreated controls (GraphPad Prism 6.0 software, San Diego, CA, USA). Data are presented as bar graphs indicating mean ± standard deviation (SD). In all cases, *p* < 0.05 was considered statistically significant.

## 3. Results

### 3.1. VAC Increases Bone Tissue Formation In Situ

**Figure 3:**
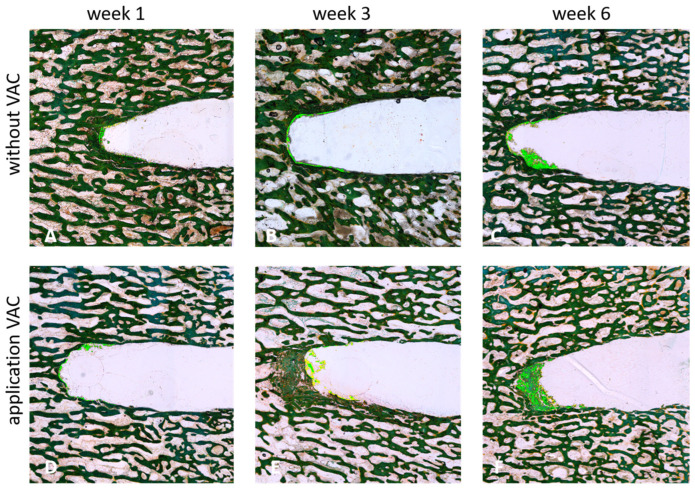
Longitudinal sections of cylindrical bone specimen after 1 (**A**,**D**), 3 (**B**,**E**) or 4 (**C**,**F**) weeks of incubation with (530 mbar) (**D**–**F**) or without (**A**–**C**) the application of VAC (Masson-Goldner staining). Newly generated bone tissue is highlighted by the light green color.

After 1, 3, or 6 weeks of applying negative pressure (530 mbar and 725 mbar), tissue growth and potential regeneration within the created defect were assessed and compared to tissue regeneration under atmospheric pressure (1013 mbar). Cross-sectional and longitudinal sections of the respective bone cylinders were examined microscopically and quantitatively evaluated (see Figure 3).

An increase in newly generated bone tissue (highlighted in light green; see Figure 3) was clearly observed over time. This effect was stronger with the application of VAC, with the highest amount of newly formed bone tissue detected after 6 weeks at 530 mbar (see Figure 3F).

**Figure 4:**
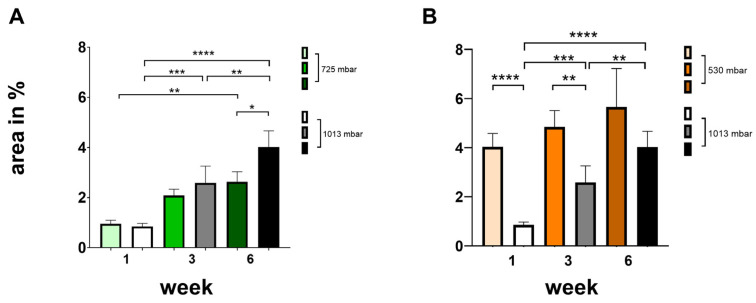
Influence of negative pressure (725 mbar: (**A**); 530 mbar: (**B**)) on bone formation [area in %] in comparison to atmospheric pressure (* *p* ≤ 0.05, ** *p* ≤ 0.01, *** *p* ≤ 0.001, **** *p* ≤ 0.0001, mean ± standard deviation, *n* = 10).

To quantify tissue growth and new bone formation within the defect, the respective regions were identified microscopically based on different wavelengths. Within the region of interest, the absolute area of newly generated bone tissue was measured in μm^2^ and calculated as a percentage (see Figure 4).

At 725 mbar, bone formation (area in %) after 1 and 3 weeks was comparable to that observed under atmospheric pressure. However, after 6 weeks of incubation, increased bone formation was evident even without negative pressure (see Figure 4A).

At 530 mbar, bone formation (area %) after 1, 3, and 6 weeks was clearly increased compared to atmospheric pressure (see Figure 4B). The effect was most pronounced after 1 week, markedly increasing by 475%. Significant increases were also observed after 3 weeks (190%) and after 6 weeks (140%), all of which were highly significant.

### 3.2. VAC Increases Mineralization in Cultured Osteoblasts

Osteoblasts derived from bone borehole grindings were successfully cultured under negative pressure (530 mbar and 725 mbar) and under atmospheric pressure (1013 mbar).

**Figure 5:**
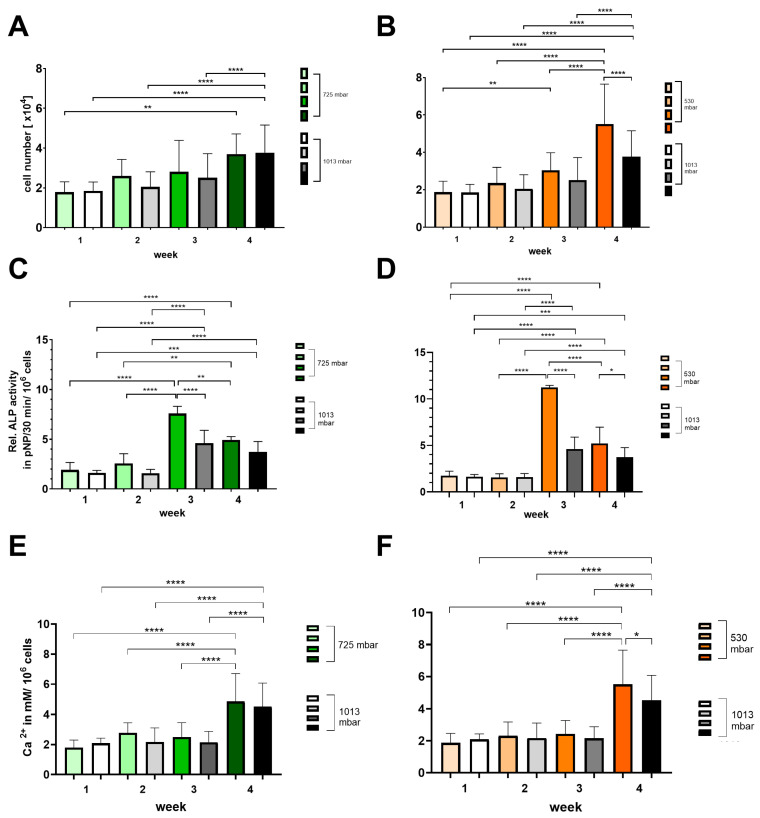
Time-dependent influence of negative pressure (725 mbar: green bars; 530 mbar: orange bars) on proliferation and osteogenic differentiation (**A**,**B**) (ALP activity: (**C**,**D**); calcium deposition: (**E**,**F**)) in comparison to atmospheric pressure (1013 mbar: black bars) (* *p* ≤ 0.05, ** *p* ≤ 0.01, *** *p* ≤ 0.001, **** *p* ≤ 0.0001, mean ± standard deviation, *n* = 10).

Growth and osteogenic differentiation were demonstrated by assessing cell number (see Figure 5A,B), alkaline phosphatase activity (see Figure 5C,D), and calcium deposition (see Figure 5E,F).

At 725 mbar, the cell counts ([1 × 10^4^]) after 1 and 4 weeks are comparable to those under atmospheric pressure, while after 2 and 3 weeks of incubation, the cell counts increased to 130% and 110%, respectively (see Figure 5A,B). In contrast, at 530 mbar, a consistent increase in cell count of up to 150% compared to atmospheric pressure was observed from week 2 onwards (see Figure 5A,B).

The ALP activity (pNP/30 min/10^6^ cells) peaked at 3 weeks for both 725 mbar and 530 mbar and then declined by week 4. Compared to atmospheric pressure, ALP activity after 3 weeks was significantly increased by 165% at 725 mbar and by 245% at 530 mbar (see Figure 5C,D).

This increase in ALP activity was accompanied by a corresponding rise in calcium content (mM/10^6^ cells) after 4 weeks. Compared to atmospheric pressure, calcium content was elevated by 110% at 725 mbar and by 120% at 530 mbar.

### 3.3. VAC Increases Expression of Osteogenic Marker Genes in Cultured Osteoblasts

**Figure 6:**
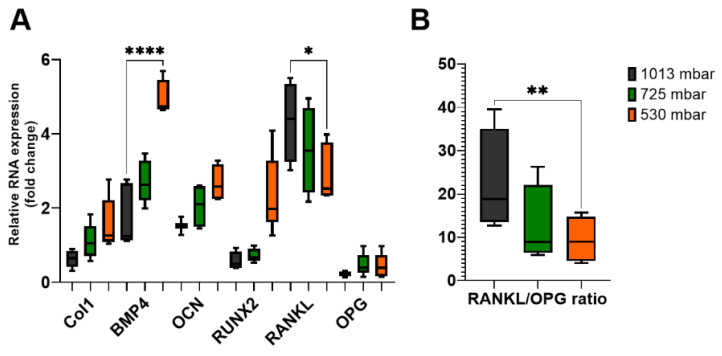
Gene expression levels of osteogenic markers (**A**) in osteoblasts treated with (725 mbar, orange bars; 530 mbar, green bars) or without VAC (1013 mbar, black bars) for 3 weeks. Reverse transcription quantitative PCR results of BMP4, Col1, OCN, RUNX2, RANKL, and OPG were normalized to GAPDH (*n* = 10). RANKL/OPG ratio (**B**) was calculated mathematically (* *p* ≤ 0.05, ** *p* ≤ 0.01, **** *p* ≤ 0.0001, mean ± standard deviation).

To elucidate the mechanisms underlying the increased mineralization following VAC treatment, we investigated the expression levels of specific osteogenic markers involved in the coupling of bone formation and resorption. After 21 days of VAC treatment, the expression levels of collagen 1 (Col1), bone morphogenetic protein 4 (BMP4), osteocalcin (OCN), and Runt-related transcription factor 2 (RUNX2) were increased (see Figure 6A).

After 3 weeks of incubation, Col1 expression increased by 175% at 725 mbar and by 250% at 530 mbar compared to atmospheric pressure. BMP4 expression increased by 155% and 280%, respectively, and OCN expression increased by 135% and 175%, respectively. For RUNX2, an increase of up to 400% was observed at 530 mbar compared to the untreated controls.

Finally, we investigated the expression levels of RANKL and OPG, which are involved in the coupling of bone formation and resorption. The RANKL/OPG ratio was also affected by VAC application. While only marginal differences were observed after 1 week, after 3 weeks, RANKL expression decreased to 82% at 725 mbar and 68% at 530 mbar compared to the untreated controls, whereas OPG expression remained nearly unchanged. This resulted in a decreased RANKL/OPG ratio in osteoblasts treated with 725 mbar (55%) and 530 mbar (41%) (see Figure 6B).

## 4. Discussion

Negative-pressure wound therapy (NPWT), commonly known as vacuum-assisted closure (VAC), is a widely used and well established treatment method. VAC therapy is also employed in traumatic wounds with bone exposure [4,5,6], although the benefits of NPWT on bone healing remain controversial. However, many clinical studies have shown that NPWT can effectively improve wound and bone healing, or at least that it does not have adverse effects on fracture healing [4,5,6,23,24,25]. In oral and maxillofacial surgery, VAC has been successfully applied in cases of poor wound conditions, such as infected osteoradionecrosis [26]. Given the complex anatomy of the maxillofacial region, where soft and hard tissue are in close proximity, it is important to understand the effects of negative pressure on different cell types, particularly bone cells. Traditionally, animal experiments—such as those using cranial bone models—have been conducted to study the effect of VAC on bone healing [27,28,29,30]. To reduce animal testing and gain more insight into oral bone regeneration under NPWT, we developed a novel approach, using bovine mandibles. Bone defect organ cultures and osteoblast cell cultures were established from bovine mandibular condylar processes samples; for the latter, bony drill grindings were used for cell culture. This approach ensures that both the bone tissue and bone cells are of identical origin, allowing the results from both culture techniques under VAC to directly complement each other.

In our bone drill organ model, we consistently observed a faster reduction of the defect using NPWT. The effect was most pronounced at 530 mbar, where up to 30% more newly formed bone tissue was generated within the drill hole compared to the control group. Similarly, Chen et al. and Zhu et al. also demonstrated accelerated bone regeneration in cranial bone defect and gap healing models in rabbits treated with VAC. Unlike our organ culture model, these studies were able to assess the influence of an additional periosteal cover on the injured bone. The presence of an intact periosteum resulted in greater bone regeneration compared cases without periosteum [27,29]. In this context, enhanced bone revascularization has been confirmed [15,31,32,33,34]. Furthermore, it is believed that NPWT stimulates bone healing by transferring mechanical load to the underlying periosteum, thereby stretching the cells [30]. In general, VAC increases the production of vascular endothelial growth factor (VEGF), microvascular density, the concentration of transforming growth factor-beta 1 (TGF-b1), platelet-derived growth factor (PDGF), and FGF2 expression [35,36,37]. At the same time, bone repair in defects or fractures is enhanced by inhibiting the degradation of gelatin and collagen [20,38,39,40] under VAC treatment [31]. Zhang et al. reported increased levels of VEGF and BMP-2 during bone healing of cranial defects in rabbits [28]. VEGF plays a crucial role in angiogenesis—a prerequisite for wound healing—and also has a key role in the differentiation and recruitment of osteoblasts and osteoclasts [20]. In another animal experiment using rats, a macroautophagy/autophagy axis involving the AMP-activated protein kinase pathway was identified, which may explain the improved bone regeneration observed [24].

In contrast to in vivo data showing that VAC influences connective tissue, blood vessels, and the periosteum, our cell culture results clearly demonstrate that osteocytes themselves are stimulated to proliferate by NPWT. Our experiments explain the success of vacuum therapy at the osteoblast level, emphasizing that mechanical stress is the most relevant factor [13,14]. Mechanical loading is a key regulator of bone modeling and remodeling [41,42]. It has also been shown that a lack of mechanical stress can significantly reduce the ability of mesenchymal stem cells to differentiate into osteoblasts [40], inhibiting the proliferation of bone marrow-derived mesenchymal stromal cells [43].

The mechanical influence on cell growth can be explained by the relationship between cell growth and cell shape [44]. Our results show that the application of NPWT to bone cells led to changes in their morphology, a finding consistent with other studies [30,45], with the strongest stimulatory effect observed at a near semi-atmospheric pressure (530 mbar). It is presumed that changes in cell shape, along with the configuration of internal cell structures such as microfilaments and microtubules, play a significant role [46]. Integrins, which are key mechanotransduction molecules linking major extracellular matrix (ECM) components to the intracellular cytoskeleton, are crucial in controlling ostoblast differentiation. Early osteoblast-specific gene expression can be influenced by mechanical loading and signaling pathways involving integrin–ECM interactions [47]. Osteoclast maturation has been shown to accelerate in mice lacking the integrin β5 subunit [48], and integrin β 5 expression is higher in osteoclasts from patients with autosomal dominant osteopetrosis type II than in those from healthy individuals [49]. Zhu et al. demonstrated that the mechanotransduction molecule integrin β5 is highly expressed after NPWT treatment [30]. The results of our study show increased ALP expression and significant improvement in mineralization in NPWT-treated cells compared to the control group, findings that are consistent with those reported by Zhu et al. [30]. We also observed a more homogeneous distribution of calcium. Calcium is essential for bone formation and plays a crucial role in bone stability [50]. In our study, cell number, ALP activity, and calcium deposition increased by up to 150%, 245%, and 120%, respectively. Furthermore, the expression of osteogenic markers such as Col1, BMP4, OCN, and RUNX2 increased by up to 250%, 280%, 175%, and 400%, respectively. These findings are in line with decreased RANKL levels (down to 68%) and a reduction in RANKL/OPG ratio to 41%, reflecting a positive impact on bone metabolism. Osteoprotegerin (OPG) functions as a decoy receptor for RANKL, preventing RANKL from binding to its receptor RANK and thereby serving as a negative regulator of osteoclastogenesis. Thus, the RANKL/OPG ratio is an essential determinant of bone mass and skeletal integrity [51,52]. In our study, VAC treatment was associated with a decreased RANKL/OPG ratio, providing a pro-osteogenic environment.

## 5. Conclusions

VAC treatment shows beneficial effects on bone regeneration. In our study, a pressure of 530 mbar exerted a more effective stimulus on the cells in vitro compared to 750 mbar. However, it is important to note that these findings are based on in vitro experiments, and additional in vivo studies are required to confirm these results. Furthermore, the development of an adapted VAC System that can be adjusted to the contours of the head and neck is urgently needed. Nevertheless, VAC has been demonstrated to be a safe and effective method. Patients with complicated maxillofacial wounds, in particular, could benefit from a tailored therapy that promotes accelerated regeneration of soft and hard tissues, resulting in a more rapid healing process. Combined with the well-known regenerative potential of soft tissue, VAC therapy could be a valuable approach for addressing the unique challenges associated with maxillofacial wounds.

## Data Availability

The data presented in this study are available on request from the corresponding author.
